# Supplementary material for: Relationship between clinical parameters and malformations in dogs diagnosed with atlanto-axial instability
Source: PLoS One. 2024 Feb 7;19(2):e0293363. doi: 10.1371/journal.pone.0293363 (PMC10849214; doi:10.1371/journal.pone.0293363)
Supplement: Attachment — Submitted filename: revision-20230822.docx [file pone.0293363.s002.docx]

1. In the Discussion, the paper has described the treatment of AAI, selection of surgical procedures, and identification. However, the correlation between AAI and body weight, as described in this paper, has no clear pertinence for the treatment and prognosis of AAI.
2. Figures 3 and 5 are not labeled in detail, preventing a better understanding of dens dysplasia and intracranial arachnoid cyst.
