# Supplementary material for: Relationship between clinical parameters and malformations in dogs diagnosed with atlanto-axial instability
Source: PLoS One. 2024 Feb 7;19(2):e0293363. doi: 10.1371/journal.pone.0293363 (PMC10849214; doi:10.1371/journal.pone.0293363)
Supplement: Attachment — Submitted filename: revision-20230822.docx [file pone.0293363.s003.docx]

1. In the Discussion, the paper has described the treatment of AAI, selection of surgical procedures, and identification. However, the correlation between AAI and body weight, as described in this paper, has no clear pertinence for the treatment and prognosis of AAI.

We modified this sentence as follow.

It is known that some AAI cases do not show postoperative neurological improvement. However, the factors that contribute to this are not known. There are also reports that dorsal decompression should be considered based on preoperative CT and MRI imaging, but no clear guidelines regarding the choice of surgical technique according to comorbidities are recognized [19, 23]. Because this is an observational study, it is unclear whether the correlation between concurrent diseases and body weight in AAI cases has clear implications for the treatment and prognosis of AAI. However, considering these reports and the results of this study, it may possible that weight, detailed preoperative imaging of concurrent diseases and assessment of postoperative neurologic improvement rates may be relevant.

2. Figures 3 and 5 are not labeled in detail, preventing a better understanding of dens dysplasia and intracranial arachnoid cyst.

We modified figures and its figure legends as you mentioned.
